# Validity and reliability of the Portuguese version of the modified Migraine Disability Assessment

**DOI:** 10.1186/s12883-021-02085-z

**Published:** 2021-02-06

**Authors:** Pedro L. Ferreira, Isabel Luzeiro, Margarida Lopes, André Jorge, Bruno Silva, Lara Ferreira

**Affiliations:** 1 Centre for Health Studies and Research of University of Coimbra/Centre for Innovative Biomedicine and Biotechnology, Faculty of Economics of University of Coimbra, Coimbra, Portugal; 2 Neurology Department of Coimbra University Hospital Centre, Faculty of Medicine of University of Coimbra, Coimbra, Portugal; 3 Neurology Department of Braga Hospital, Braga, Portugal; 4 Neurology Department of Coimbra University Hospital Centre, Coimbra, Portugal; 5 Centre for Health Studies and Research of University of Coimbra/Centre for Innovative Biomedicine and Biotechnology, University of Algarve, School of Management, Hospitality and Tourism, Faro, Portugal

**Keywords:** Patient reported outcomes, Quality of life, Migraine, mMIDAS-P, Productivity, Disability

## Abstract

**Background:**

Migraine Disability Assessment Scale (MIDAS) is a useful tool to measure headache-related disability. Modified MIDAS with 4-week recall period reduces recall bias and improves accuracy of the results. This study aimed at validating mMIDAS in Portuguese.

**Methods:**

Studied population consisted of adult migraine patients attending a headache outpatient clinic. Reliability was assessed by internal consistency and reproducibility in a 3-week test-retest. Content validity was evaluated by two expert panels. Construct validity was tested by comparing mMIDAS-P index in socioeconomic and clinical patient groups and scale unidimensionality was evidenced by factor analysis. Criterion validity was tested using EQ-5D-5L and HADS.

**Results:**

Ninety-two patients, 88% female, mean age of 44 years, participated. They had, in average, 9.7 headache days in previous month, pain averaging 7.5/10. About 69.9% were on a migraine prophylactic treatment, and 42.4% had severe disability; 29.4 and 13.0% showed, respectively, moderate/severe anxiety and depression. Content validity showed that mMIDAS-P is simple and clinically useful. It did not show to be determined by patient’s sociodemographic characteristics and it was correlated with depression scale and EQ-5D-5L. Test-retest demonstrated high reproductive reliability and good internal consistency.

**Conclusion:**

mMIDAS-P is valid and reliable. We strongly recommend it for clinical and research use.

**Supplementary Information:**

The online version contains supplementary material available at 10.1186/s12883-021-02085-z.

## Background

Migraine is an episodic primary headache syndrome, the most common type of headache worldwide, affecting 1 out of 10 people [1]. Although pain is the most commonly reported symptom, migraine is associated with non-painful symptomatology, including aura, prodrome and postdrome symptoms, which can be as disabling as pain itself, with all its spectrum of manifestations. Migraine is a highly disabling condition, with impact at the social, family and professional levels, profoundly affecting patient quality of life (QoL). In Portugal, headache disorders are the third most disabling health problem with over 2 million people suffering from migraine in the country; its prevalence is 18.1%, all ages [2].

A useful tool to measure this disability is the Migraine Disability Assessment Scale (MIDAS), developed after 1998 by Stewart and colleagues [3–6], aimed to improve the communication between care providers and migraine patients. It was developed from the Headache Impact Questionnaire (GImQ) to measure headache severity, excluding the pain dimension, to obtain an easier way to compute a global score [7]. MIDAS has been largely used to evaluate the impact of migraine and has been proven simple to use, consistent, highly reliable, and correlating well with physicians’ clinical judgments in several studies, not only in USA and UK English [6, 8], but also in many other languages. Among such languages, we may cite, by alphabetic order, Arabic [9], Chinese [10], French [11], German [12], Greek [13], Hindi [14], Italian [15], Japanese [16], Malay [17], Persian [18], Portuguese-Brazil [19], Spanish [20], Thay [21], and Turkish [22]. MIDAS also proved to be useful to identify adequate treatments and to stratify them based on the level of patient disability [9, 23].

Recently, a minor modification was performed in the original MIDAS questionnaire: the recall time was changed from 3-months to 4-weeks [24]. This modification was expected to reduce recall bias and improve accuracy of the results, and termed the questionnaire mMIDAS.

Portuguese is the ninth most spoken language in the world, with 234 million speakers and, although MIDAS had previously been translated into European Portuguese, it had not yet been validated in this language. The authors endeavoured to apply this translated version of MIDAS on a migraine Portuguese population, to evaluate the reliability, internal consistency, and validity of the Portuguese mMIDAS (mMIDAS-P) questionnaire.

## Methods

### Study design

This study aimed at validating mMIDAS and studying the QoL of a sample of migraine patients. Under no circumstances, it interfered with health professionals’ decisions concerning the most appropriate medical approach for each patient. Before data collection, all participants filled in a written informed consent and this study received the approval from the Ethics Commission of the Coimbra University Hospital.

### Participants

The study was conducted between September 25th and December 6th^th^ 2019 at the Neurology Department of Coimbra University Hospital. Studied population consisted of consecutive adult patients observed in a headache outpatient clinic. These were evaluated and included in the study by a clinician if older than 18 years, with chronic or episodic migraine, with ability to give consent to participate in the study, and if able to read and write Portuguese for self-completion of questionnaires. Excluded were unstable patients or with uncontrolled symptoms and considered by clinicians as unable to fill the measures, cognitively affected, and those who could not understand Portuguese. The questionnaire was completed during the consultation.

### Measures

Besides mMIDAS, we applied a generic QoL instrument (EQ-5D-5L), two questions about satisfaction with life and social support, a specific psychological instrument to measure anxiety and depression (HADS); and a short socioeconomic set of questions.

mMIDAS is a self-administered questionnaire that contains seven questions about the headache a patient had in the previous month [3–6]. The first five questions assess the impact of migraine on three domains of daily activity: two questions for paid work or schoolwork, two questions for household work, and one question for family, social and leisure activities. The two questions for each of the first two groups assess, respectively, the number of days off due to headache, and the number of days in which the productivity was reduced by half or more.

The sixth and seventh additional questions, for the clinicians’ benefit, count the frequency of headache days in the previous month, and the average intensity of the headache attacks, in a 0 (no pain at all) to 10 (pain as bad as it can be) scale.

mMIDAS index is derived from the sum of the answers on the first five questions. This score defines patients in four categories of headache disability: little/none disability if the score is between 0 and 5; mild disability if between 6 and 10; moderate disability if between 11 and 20; and severe disability if greater than 20 [4–6].

The original version of MIDAS is available on the National Headache Foundation website and is presented in the Supplementary file 1. A first Portuguese non-validated translation of mMIDAS has been provided by the author through Mapi Research Trust (see Supplementary file 2). Therefore, before implementing this measure, we decided to validate its contents by clinical reviews with Portuguese neurologists and by cognitive debriefings with patients. We also tested its reliability, as well as its construct and criterion validity.

EQ-5D-5L is a short generic QoL preference measure that allows generating an index representing the value assigned to an individual’s health status. It was developed by the EuroQoL Group in 1987 and it is currently composed of a descriptive system and a visual analogue scale (VAS). The descriptive system represents health in five dimensions: mobility, self-care, usual activities, pain/discomfort and anxiety/depression. Each of these dimensions has five associated severity levels and a weighted scoring procedure to create an EQ-5D-5L index score [25].

VAS has the appearance of a thermometer (vertical line with about 20 cm), whose scale ranges from 0 (worst imaginable health status) to 100 (best imaginable health status), with the individual having directly to mark what value s/he attributes to her/his current health status.

In this study, we have used the Portuguese version approved by the EuroQoL Group [26].

To measure the satisfaction with life we asked the patient whether s/he agreed with the sentence “I am happy with my life”, in a 5-point scale from ‘completely disagree’ to ‘completely agree’. The social support question refers to the expected number of individuals the patient would be able to ask for help in case of need.

The Hospital Anxiety and Depression Scale (HADS) is composed by 14 items, grouped in two subscales separately scored and measuring, respectively, anxiety and depression. Each item is answered in a 4-point Likert scale from 0 to 3 and it takes approximately 2–5 min to be completed [27]. For each subscale, a score between 0 and 7 is considered normal, between 8 and 10 mild, between 11 and 14 moderate and between 15 and 21 severe.

In this study, we have used the Portuguese validated version [28].

The sociodemographic information included gender, age, marital status, years of education and employment status.

We also asked physicians to provide clinical information about patient’s health status. This included age of migraine diagnosis, time of follow-up, headache frequency and characteristics, use of prophylactic treatment and types of drugs used in the past to prevent headache, as well as acute headache treatment. At last, we also collected information on patients’ comorbidities.

Statistically, one first decision we had to make was related to the use of non-parametric statistics, depending on whether mMIDAS-P index follows a normal distribution.

Therefore, we firstly raised the following hypothesis:

- H_1_: The distribution of mMIDAS-P index is normal

### Reliability

Reliability was assessed through intertemporal stability and internal consistency. To test the intertemporal stability, we used the intraclass correlation coefficient (ICC). To avoid recall bias, eligible patients filled mMIDAS-P in two consecutive moments 3 weeks apart, the same interval used by the authors in their original validation study [4, 5], as well as in some other countries. As defended by Koo, an ICC score lower than 0.50 corresponds to a weak correlation, between 0.50 and 0.75 to a moderate correlation, between 0.75 to 0.90 to a good correlation and a score higher than 0.90 corresponds to an excellent correlation [29]. We also looked at the correlation factor between each item of mMIDAS-P and the whole scale.

The internal consistency was measured by the Cronbach’s alpha coefficient, where recommended values should be between 0.70 and 0.90 [30]. For this study, two hypotheses were raised:

- H_2_: mMIDAS-P shows good intertemporal stability.
- H_3_: mMIDAS-P shows good internal consistency.

### Validity

We followed the internationally defined methodology to validate QoL measures. Under this framework, we tested content, construct and criterion validity [30]. As a translated Portuguese version already existed, we started by asking for a clinical revision performed by two neurologists, and a cognitive debriefing with 10 patients to guarantee the content validity, i.e., the relevance of the mMIDAS-P items. We formulated the following hypothesis:

- H_4_: mMIDAS-P is well accepted by clinicians and patients, not showing ambiguities, redundancies or lack of contents.

Construct validity, addressing the theoretical concepts behind the measurement instrument, encompassed the structural validity and hypothesis testing [30]. Structural validity was tested by exploratory factor analysis based on principal components estimates, having previously applied the sampling adequacy through the measure of sample adequacy Kaiser-Meyer-Olkin (KMO) and by Bartlett’s test of sphericity. A KMO smaller than 0.50, between 0.50 and 0.60 or between 0.60 and 0.70 is considered, respectively, unacceptable, poor or fair. Scores between 0.70 and 0.80, between 0.80 and 0.90 or higher than 0.90 are considered, respectively, average, good or very good [31]. Bartlett sphericity test should have an associated significance of < 0.001. For the selection of the number of factors, we followed the Kaizer criterion for eigenvalues greater than 1.

For the hypothesis testing, we formulated several hypotheses with known-groups or subsamples based on sociodemographic and clinical variables. Should mMIDAS-P index be normally distributed, Student’s t-test was to be used for two independent variables and ANOVA for more than two independent variables. Should mMIDAS-P index be not normally distributed, the nonparametric Kolmogorov-Smirnov and Kruskal-Wallis tests were to be used. The following hypotheses were then defined:

- H_5_: The structure of mMIDAS-P maintains the unidimensionality.
- H_6_: mMIDAS-P scores are dependent from sociodemographic variables.
- H_7_: mMIDAS-P scores are dependent from clinical variables.

Criterion validity was assessed by comparing mMIDAS-P index with the ones obtained by HADS and EQ-5D-5L, considered as gold standard references. We also compared mMIDAS-P index with the results from the two additional questions of mMIDAS questionnaire. The statistical rule used was mainly based on Spearman’s correlation coefficient, but Pearson’s correlation was also computed to detect the role of possible outliers. As defined by Cohen, correlations smaller than 0.30 are considered weak, between 0.30 and 0.50 are moderate, and higher than 0.50 are considered strong [32].

By comparing mMIDAS-P with HADS and EQ-5D-5L, we expected to detect similarities and differences between dimensions, such as depression [33] and migraine pain [34]. By comparing with the sixth and the seventh questions, we expected a positive correlation with headache frequency, measured by the number of days with headache, and with the headache intensity, measured by the average intensity of these headaches. Therefore, we tested the following hypotheses:

- H_8_: mMIDAS-P index correlates positively with the number of days with headache.
- H_9_: mMIDAS-P index correlates positively with the average intensity of headache.
- H_10_: mMIDAS-P index correlates with HADS dimensions anxiety and depression.
- H_11_: mMIDAS-P index correlates with both EQ-5D-5L index and VAS.

## Results

### Sample

The sample was composed by 92 patients. Socioeconomic characteristics and impact on QoL of patients are presented in Table 1. Table 2 shows some of the clinical headache characteristics.

**Table 1:** Sociodemographic characteristics of the sample (*n* = 92)

Variable	Value	N	%
Gender	Female	81	88.0
	Male	11	12.0
Age (years)	19–39	31	33.7
	40–49	33	35.9
	50–78	28	30.4
	Min- max	19–78	
	Mean ± standard deviation	44.0 ± 11.9	
	Median	44	
Marital status	Single	19	20.7
	Married or living together	61	66.3
	Widowed/divorced/separated	12	13.1
Years of education	0–4	4	4.4
	5–9	19	20.6
	10–12	30	32.6
	> 12	39	42.4
Employment status	Employed	68	73.9
	Unemployed/student	14	15.2
	Other	10	10.9
mMIDAS index	Min – max	0–126	
	Mean ± standard deviation	28.5 ± 29.7	
	25th percentile	7	
	50 ^th^ percentile	16	
	75 ^th^ percentile	38	
	Little or no disability (0–5)	15	16.3
	Mild disability (6–10)	14	15.2
	Moderate disability (11–20)	24	26.1
	Severe disability (> 20)	39	42.4
Migraine frequency (mMIDAS-6)	Min-max	0–30	
	Mean ± standard deviation	9.78 ± 8.9	
	Median	6	
How painful were the headaches, 0–10 (mMIDAS-7)	Min-max	0–10	
	Mean ± standard deviation	7.5 ± 1.9	
	Median	8	
Quality of life (EQ-5D-5L - Index)	Min-max	5–100	
	Mean ± standard deviation	82.0 ± 19.7	
Quality of life (EQ-5D-5L - VAS)	Min-max	30–100	
	Mean ± standard deviation	73.4 ± 18.2	
Anxiety (HADS)	Normal (0–7)	40	43.5
	Mild (8–10)	25	27.1
	Moderate (11–14)	17	18.5
	Severe (15–21)	10	10.9
	Min-max	0–18	
	Mean ± standard deviation	8.0 ± 3.9	
	Median	8	
Depression (HADS)	Normal (0–7)	57	62.0
	Mild (8–10)	23	25.0
	Moderate (11–14)	8	8.7
	Severe (15–21)	4	4.3
	Min-max	0–18	
	Mean ± standard deviation	6.2 ± 4.2	
	Median	6	
I am happy with my life	Totally disagree	3	3.2
	Disagree	18	19.6
	Neutral	23	25.0
	Agree	38	41.3
	Totally agree	10	10.9
Number of people to ask for help	None	5	5.4
	1–2 persons	29	31.5
	3–5 persons	39	42.4
	6 or more persons	19	20.7

**Table 2:** Clinical characteristics of the sample (*n* = 92)

Variable	Value	N	%
Age of migraine diagnosis (years)	Min-max	2–76	
	Mean ± standard deviation	31.2 ± 14.7	
	< 20	27	29.3
	21–39	39	42.4
	≥ 40	26	28.3
Time on consultation (years)	Min-max	0–37	
	Mean ± standard deviation	3.8 ± 5.6	
	< 1	36	39.2
	1–3	27	29.3
	≥ 4	29	31.5
Frequency of headaches in previous month	Min-max	0–30	
	Mean ± standard deviation	8.9 ± 4.5	
Prophylactic treatment	Now, continued	41	49.4
	Now, not previously	17	20.5
	Not now, but previously yes	18	21.7
	Never	7	8.4
Previous prophylactic treatment	Yes	59	71.1
	No	24	28.9
Drugs prescribed	Antiepileptic	50	54.3
	Antidepressant	48	52.2
	Beta-Blockers	34	36.9
	Botulinum Toxin A	1	12.0
	Calcium Blockers	7	7.6
	Non-Steroidal anti-Inflammatory	4	1.4
	Neuromodulation Devices	1	1.1
	Invasive Treatments	1	1.1
	Other	10	10.9
Acute treatment for migraine	Yes	89	97.8
	No	2	2.2
Acute therapeutics usually followed	Analgesics	84	91.3
	Trypanoes	44	47.8
	Antiemetics	15	16.3
	Opioids	6	6.5
	Ergotaminicos	5	5.4
	Corticosteroids	2	2.2
	Other	1	1.1
Other comorbidities	Yes	18	20.9
	No	68	79.1

Ninety-two patients, 88% female, with a mean age of 44 years participated. A large percentage was married or lived together (66.3%), were employed (73.9%), and a fourth had less than 9 years of education. This sample is representative to all eligible migraine patients seen by the Neurology Department of Coimbra University Hospital.

In what concerns the measure of disability due to migraine, 68.5% of the patients suffered from moderate to severe disability and the median score was 16. Migraine frequency in previous month was 9.78 times (median = 6). In a scale from 0 to 10, the average intensity of headache was 7.5 (median = 8).

In general, the QoL self-assessed by patients was reported as 82.0 from EQ-5D-5L index, and 73.4 from VAS. Patients also showed normal to mild anxiety (median 8) and normal depression (median 6). Moreover, 10.9% had severe anxiety and 4.3% were severely depressed.

Concerning the sentence ‘I am happy with my life’, 52.2% agreed and 22.9% disagreed. In the loneliness question, 63.1% declared having at least three persons to ask for help, if needed.

Regarding the clinical variables, patients had their migraine diagnosis when they were about 31 years of age, had a follow-up of about 3.8 years, and had had in average 8.9 headaches in previous month. About 70% were submitted to a prophylactic treatment, the majority of them with antiepileptic (54.3%) and antidepressant drugs (52.2%). In crisis, 97.8% of patients followed an acute treatment, especially using analgesics (91.3%) and tryptans (47.8%). In general, the large majority of patients (79.1%) did not report any other comorbidity.

As mentioned before, we firstly tested whether the variable mMIDAS-P index was normally distributed (H_1_). Following the results of the one-sample Kolmogorov-Smirnov test (test statistic = 0.225; *p* < 0.001), we decided to reject the null hypothesis, i.e., we assumed a non-normality of the mMIDAS-P index.

### Reliability

Table 3 reveals the ICC scores obtained from a 3-week test-retest on 30 patients, without any dropout. It shows an overall mMIDAS-P index highly reliable (ICC = 0.903), with partial scores between good (0.891) and excellent (0.997).

**Table 3:** ICC for test-retest study

Because of headaches, during last month,	Test (***n*** = 92)		Retest (***n*** = 30)			Correlation
	Mean	Median	Mean	Median	ICC	with MIDAS-P
[1] # missed days of work or school	2.07	0.0	1.60	0.0	0.997^2^	0.697^1^
[2] # days with productivity reduced by half or more	7.90	5.0	8.50	4.5	0.957^2^	0.828^1^
[3] # missed days of household work	5.64	2.0	9.03	3.0	0.981^2^	0.749^1^
[4] # days with household work productivity reduced by half or more	9.47	5.5	9.60	6.0	0.950^2^	0.853^1^
[5] # missed days of family, social or leisure activities	3.54	2.0	2.53	1.0	0.891^2^	0.564^1^
MIDAS- P index	28.5	16.0	29.2	20.0	0.903^2^	
[6] # days with headache	9.78	6.0	10.5	10.0	0.832^2^	
[7] In a 0–10 scale, on average, how painful were these headaches	7.49	8.0	8.07	8.0	0.780^2^	

^1^ *p* < 0.01 ^2^ *p* < 0.001

Cronbach’s alpha indicator of internal consistency was 0.793, within the recommended set of values. Therefore, we did not reject hypotheses H_2_ and H_3_.

Furthermore, the correlations between mMIDAS-P index and each of the five components revealed scores from moderate (mMIDAS5) to good (mMIDAS4).

### Validity

To assess semantic and cultural validity of mMIDAS-P version provided by Mapi, we asked two neurologists to perform clinical reviews. Very few and minor changes were proposed and immediately accepted by the research team. On the other hand, two cognitive debriefing meetings were performed with 10 migraine patients (two men; eight women), who filled mMIDAS-P in 2.9 ± 1.5 min (median = 2.0 min). Patients did not evidence difficulties in understanding the measure nor did they find redundancies or ambiguities in the questions. In addition, the scores were very easy to be computed. We did not reject H_4_.

To test construct validity and the factor structure of mMIDAS-P index, exploratory factor analysis was applied to the database of 92 responses. This analysis allowed us to guarantee the unidimensionality defined by the author, with 55.6% of explained variance and the factor loadings as presented in Table 4. The KMO test obtained was 0.733 and the Bartlett sphericity test had an associated significance (< 0.001), which allowed us to pursue with the analysis.

**Table 4:** Factor analysis scores

Because of headaches, during last month,	Factor loadings
[1] # missed days of work or school	0.738
[2] # days with productivity reduced by half or more	0.821
[3] # missed days of household work	0.746
[4] # days with household work productivity reduced by half or more	0.839
[5] # missed days of family, social or leisure activities	0.548

Even when including all seven mMIDAS questions in the factor analysis, the unidimensionality could not be rejected; however, question mMIDAS7 had a very low communality (0.196) and the total percent of variance decreased a little bit to 52.6%. We concluded that it is better to maintain only the five first questions as part of the total mMIDAS-P score (H_5_).

Construct validity was tested by comparing mMIDAS-P index with patients’ sociodemographic characteristics (H_6_). As presented in Table 5, mMIDAS-P did not show to be determined by patient’s sociodemographic gender, age or education variables.

**Table 5:** mMIDAS scores for different levels of sociodemographic and clinical variables

Variable	Value	n	Mean	Comparisons	mMIDAS Grade Disability			
				Test Statistic (sig)	None/little/mild	moderate	severe	χ^**2**^ (sig)
Gender^a^	Female	81	28.7	0.940	86.2%	79.2%	94.9%	3.617 (*p* = 0.164)
	Male	11	25.5	(*p* = 0.340)	13.8%	20.8%	5.1%	
Age^b^	19–39	31	20.3	3.202	44.8%	37.5%	23.1%	5.335 (*p* = 0.255)
	40–49	33	33.8	(*p* = 0.202)	34.5%	25.0%	43.6%	
	50–78	28	31.1		20.7%	37.5%	33.3%	
Marital status^a^	Single	19	13.7	1.666	31.0%	33.3%	5.1%	9.998 (*p* = 0.007)
	Non-single	73	32.3	(*p* = 0.008)	69.0%	66.7%	94.9%	
Years of education^a^	≤ 12 years	53	24.0	1.236	72.4%	54.2%	48.7%	3.982 (0.137)
	> 12 years	39	34.9	(*p* = 0.094)	27.6%	45.8%	51.3%	
Age of migraine diagnosis (years)^b^	≤ 20	27	21.0	1.025	34.5%	25.0%	28.2%	1.095 (*p* = 0.895)
	21–39	39	28.8	(*p* = 0.599)	37.9%	50.0%	41.0%	
	≥ 40	26	35.8		27.6%	25.0%	30.8%	
Time of follow-up (years) ^b^	< 1	36	32.3	0.578	34.5%	37.5%	43.6%	1.914 (*p* = 0.752)
	1–3	27	26.4	(*p* = 0.749)	31.0%	37.5%	23.1%	
	≥ 4	29	25.6		34.5%	25.0%	33.3%	
Previous prophylactic treatment^a^	Yes	59	32.0	0.896	64.0%	61.9%	81.1	3.270 (*p* = 0.195)
	No	24	24.2	(*p* = 0.399)	36.0%	38.1%	18.9	
Other comorbidities^a^	Yes	18	25.8	0.512	22.2%	17.4%	22.2%	0.238 (*p* = 0.888)
	No	68	29.2	(*p* = 0.956)	77.8%	82.6%	77.8%	

^a^Independent-samples Kolmogorov-Smirnov test; ^b^Independent-samples Kruskal-Wallis test

However, single patients tend to have a lighter impact of their migraine.

In what concerns the clinical variables age of migraine diagnosis, time of follow-up, previous prophylactic treatment, and other comorbidities, mMIDAS-P also showed not to be determined by any level of these variables (H_7_).

To test criterion validity, we correlated mMIDAS-P index with the number of days with headache [mMIDAS6]. The result of Spearman’s correlation was 0.793 (*p* < 0.01), evidencing a positive significant correlation (H_8_). We also compared mMIDAS-P index with the average intensity of headaches (mMIDAS7). In our sample, the correlation between these two variables was 0.300 (*p* = 0.004), also evidencing a positive significant correlation (H_9_) (Table 6).

**Table 6:** Correlation between mMIDAS scores, QoL variables and headache characteristics

	Variable	Pearson r	Spearman rho
Headaches	# days with headache	0.836 (*p* < 0.001)	0.792 (*p* < 0.001)
	Average intensity	0.346 (*p* = o.001)	0.300 (*p* = 0.004)
HADS	Anxiety	0.181 (*p* = 0.084)	0.160 (*p* = 0.128)
	Depression	0.507 (*p* < 0.001)	0.339 (*p* = 0.001)
EQ-5D-5L	Mobility	0.183 (*p* = 0.080)	0.104 (*p* = 0.326)
	Self-care	0.306 (*p* = 0.003)	0.157 (*p* = 0.136)
	Usual activities	0.648 (*p* < 0.001)	0.512 (*p* < 0.001)
	Pain/discomfort	0.507 (*p* < 0.001)	0.408 (*p* < 0.001)
	Anxiety/depression	0.537 (*p* < 0.001)	0.422 (*p* < 0.001)
	Index	−0.675 (*p* < 0.001)	−0.474 (*p* < 0.001)
	VAS	−0.457 (*p* < 0.001)	−0.442 (*p* < 0.001)

The EQ-5D-5L dimensions with higher correlation scores with mMIDAS-P were pain/discomfort (0.422; *p* < 0.001) and anxiety/depression (0.422; *p* < 0.001). mMIDAS-P also showed to be correlated with HADS depression scale (0.568; *p* < 0.001) (H_10_) and with both EQ-5D-5L index (− 0.474; *p* < 0.001) and VAS (− 0.442; *p* < 0.001) (H_11_).

## Discussion

mMIDAS index is based on answers about disability associated to migraine, in previous month. This disability is measured in terms of missed days of activity and days with reduced productivity, considering productivity at work or school, household work and non-work (family, social, leisure) activity. With this measure, we assume that a very severe headache may result in missing more than one of these activities, and so, a single day of headache may correspond to more than one point in the index [6, 10]. On the other hand, results show that mMIDAS-P index was skewed distributed towards high values, as the 25th, 50th and 75th percentiles were, respectively, 5 (little/mild disability), 16 (moderate disability) and 38 (severe disability).

Sociodemographic variables in our study were similar to those in other MIDAS validations: higher prevalence of females, patients in the 40–49 age group, employed, with a better QoL, with mild anxiety, normal depression, and an average of about 10 episodes of headache in previous month, with an average severity of 7.5 in a 0–10 scale. In what concerns happiness with life, more than half of the patients declared being satisfied and, in general, with enough social support if needed [4, 5].

We found evidence that migraine patients are more willing to miss household work than family, social or leisure activities, being the employment or school work the one they are less willing to miss. This can probably be explained by the fact that, because paid or school work are considered as more stable activities, they are more easily remembered by patients, as also hypothesized by Hung and colleagues [10]. Also, the number of days in which the activities are reduced by half or more were always higher than the number of full days missed, which may inform us that some patients still maintain their work even suffering from pain [10].

The average mMIDAS-P index was 28.5 and the range varied from 0 to 126. Regarding the question mMIDAS7 addressing the average intensity of headache pain, we obtained (Table 3) a lower reliability (ICC = 0.780). However, we did not feel any need to modify the question as happened in Hindi [14] and in Turkish [22] versions. Likewise, we decided to maintain mMIDAS6 as it was, and not use intervals instead of fixed number of days, as proposed by the French version [11].

Even recognizing that older studies used Pearson’s or Spearman’s correlations to test intertemporal stability and newer studies used ICC, the reliability scores obtained in this study (ICC = 0.821 and alpha = 0.793) are comparable with the ones reported by other countries’ studies. Table 7 presents the reliability scores of validations in other languages.

**Table 7:** Comparable reliability score with the original and some MIDAS versions

Country	Language	Source	Test-retest reproducibility	Internal consistency
Portugal	Portuguese		0.90 ^b^	0.80
Lebanon	Arabic	14	0.99 ^b^	0.81
Taiwan	Chinese	15	0.79 ^a^	0.67
USA	English	8	0.80 ^a^	0.76
UK	English	8	0.83 ^a^	0.73
Canada	French	16	0.84 ^b^	NA
Switzerland	German	17	0.99 ^b^	0.69
Greece	Greek	18	0.82 ^b^	0.71
India	Hindi	19	0.94 ^a^	> 0.90
Italia	Italian	20	0.81 ^a^	0.74
Japan	Japanese	21	0.83 ^a^	0.82
Malaysia	Malay	22	0.87 ^a^	0.84
Iran	Persian	23	0.71 ^a^	0.80
Brasil	Portuguese	24	0.77 ^a^	0.70
Spain	Spanish	25	0.81 ^b^	0.80
Thailand	Thai	26	0.76 ^b^	0.91
Turkey	Turkish	27	0.68 ^a^	0.73

^a^Pearson’s/Spearman’s correlation coefficient; ^b^Interclass correlation coefficient

NA not available in the paper

Also, the correlation factors between each mMIDAS-P item and the whole scale gave correlations between 0.564 (moderate) and 0.853 (good), all of them significant (*p* < 0.01). Internal consistency Cronbach’s alpha is very comparable with the values obtained in the original version and from other languages.

In what concerns validity, mMIDAS-P results are, in general, comparable with other validation studies, even with different methodology. UK and USA [5], Japan [16] and Thailand [21], compared their results with 90-day diary measures patients were supposed to fill; some studies did it with HIT-6 as Hindi version [14], and some others with SF-12, SF-36 or RAND-36, as the Persian [18] or Greek [13] versions. However, as far as we know, our version is the first one comparing MIDAS scores with psychological indicators and preference-based QoL measures.

The construct validity confirmed the unidimensionality of mMIDAS-P, as in the original version and the results from Shaik [17] and Mourad [9]. The Spanish version [20], however, in a population of young university students, presented a 2-factor structure, one composed by engagement in activities (items 1, 3 and 5), and the other corresponding to the performance at work (items 2 and 4).

Comparing mMIDAS-P index with scores obtained in known-groups, we found no significant differences among the several levels of patients’ characteristics, namely gender, age and education, as well as clinical variables age of migraine diagnosis, time of follow-up, previous prophylactic treatment, and other comorbidities.

Criterion validity was tested by comparing the mMIDAS-P index with the two extra mMIDAS items, with self-reported anxiety and depression from HADS and with QoL scores provided by EQ-5D-5L. Portuguese mMIDAS index showed a positive and significant correlation with both extra items, with anxiety index, especially with pain/discomfort and anxiety/depression of EQ-5D-5L. It should be noted that mMIDAS focusses on symptoms related with migraine.

One limitation of this study may derive from the sample size. We collected data from 92 migraine patients, very close from our initial goal of having 100 patients. However, considering the COSMIN checklist [35] and the non-existed missing values, our sample size may be considered very good for the measurement properties we used to validate mMIDAS.

A second possible limitation may lie in the patients’ characteristics of our sample. These patients were recruited from an ambulatory department’s migraine consultation of a general hospital. Considering the mMIDAS index, circa than one third of them had, at most, a mild migraine. We are confident that our range is representative from the actual distribution of migraine patients from the general population.

A third issue we should address is that, when we compare mMIDAS scores with those obtained from more generic measures we may run the risk of comparing with a wider range of symptoms. However, migraine, when exists, usually dominates all other sources of discomfort, and so, we think that the comparisons made were appropriate.

## Conclusion

This study supports the use of the Portuguese version of mMIDAS (mMIDAS-P) is a valid and reliable instrument to evaluate disability caused by migraine. Our results are consistent with the original version as well as with other validation studies.

It is suitable for use in clinical practice as a decision aid tool to improve health care strategies centered on migraine patients. It allows the assessment of the impact on disability and improves the relationship and the communication between physician and patient [36]. It has also shown to be well accepted by migraine patients.

## Supplementary Information

**Additional file 1.**

**Additional file 2.**

## Data Availability

The dataset used and/or analysed during the current study is available from the corresponding author on reasonable request.

## Abbreviations

EQ-5D-5L: Euroqol 5dimensions 5 levels
GImQ: Headache Impact Questionnaire
HADS: Hospital Anxiety and Depression Scale
ICC: Intraclass Correlation Coefficient
KMO: Kaiser-Meyer-Olkin
MIDAS: Migraine Disability Assessment Scale
mMIDAS: Modified Migraine Disability Assessment Scale
mMIDAS-P: Portuguese mMIDAS
QoL: Quality of life
VAS: Visual Analogue Scale
