# Supplementary material for: Validity and reliability of the Portuguese version of the modified Migraine Disability Assessment
Source: BMC Neurol. 2021 Feb 6;21:58. doi: 10.1186/s12883-021-02085-z (PMC7866748; doi:10.1186/s12883-021-02085-z)
Supplement: Supplementary file 1 — Additional file 1. [file 12883_2021_2085_MOESM1_ESM.pdf]

The Migraine Disability Assessment Test

The **MIDAS** (Migraine Disability Assessment) questionnaire was put together to help you measure the impact your headaches have on your life. The information on this questionnaire is also helpful for your primary care provider to determine the level of pain and disability caused by your headaches and to find the best treatment for you.

INSTRUCTIONS

Please answer the following questions about ALL of the headaches you have had over the last 3 months. Select your answer in the box next to each question. Select zero if you did not have the activity in the last 3 months. Please take the completed form to your healthcare professional.

_____ 1. On how many days in the last 3 months did you miss work or school because of your headaches?

_____ 2. How many days in the last 3 months was your productivity at work or school reduced by half or more because of your headaches? (Do not include days you counted in question 1 where you missed work or school.)

_____ 3. On how many days in the last 3 months did you not do household work (such as housework, home repairs and maintenance, shopping, caring for children and relatives) because of your headaches?

_____ 4. How many days in the last 3 months was your productivity in household work reduced by half of more because of your headaches? (Do not include days you counted in question 3 where you did not do household work.)

_____ 5. On how many days in the last 3 months did you miss family, social or leisure activities because of your headaches?

_____ Total (Questions 1-5)

What your Physician will need to know about your headache:

_____ A. On how many days in the last 3 months did you have a headache? (If a headache lasted more than 1 day, count each day.)

_____ B. On a scale of 0 - 10, on average how painful were these headaches? (where 0=no pain at all, and 10= pain as bad as it can be.)

Scoring: After you have filled out this questionnaire, add the total number of days from questions 1-5 (ignore A and B).

MIDAS Grade	Definition	MIDAS Score
I	Little or No Disability	0-5
II	Mild Disability	6-10
III	Moderate Disability	11-20
IV	Severe Disability	21+

If Your MIDAS Score is 6 or more, please discuss this with your doctor.

© Innovative Medical Research, 1997
© 2007, AstraZeneca Pharmaceuticals, LP. All Rights reserved.
