# Supplementary material for: Validity and reliability of the Portuguese version of the modified Migraine Disability Assessment
Source: BMC Neurol. 2021 Feb 6;21:58. doi: 10.1186/s12883-021-02085-z (PMC7866748; doi:10.1186/s12883-021-02085-z)
Supplement: Supplementary file 2 — Additional file 2. [file 12883_2021_2085_MOESM2_ESM.pdf]

Mapi™
Research Trust

This translation was provided by the Author to Mapi Research Trust for distribution. Mapi has no information on the validity of this translation which cannot be considered as validated for commercial use.

It should be noted that Mapi was not involved in the linguistic validation process of this language version. For this reason, Mapi will not supply any translation certificates or supporting documentation.

For any information or questions, please submit a request in the ePROVIDE system.

Thank you !

QUESTIONÁRIO MIDAS

Escala para avaliação da incapacidade causada pela enxaqueca.

Instruções:
Por favor, responda às seguintes perguntas sobre TODAS as cefaleias que teve nos últimos 3 meses. Escreva a sua resposta a seguir a cada pergunta. Escreva 0 (zero) se não efectuou a actividade referida nos últimos 3 meses. (Se necessário, recorra a um calendário).

Nome do doente: ______________________

Data: ______/______/__________

1. Em quantos dias nos últimos 3 meses teve de faltar à escola ou ao emprego devido às cefaleias? |__|__|

2. Quando ainda conseguiu ir à escola ou trabalhar, em quantos dias nos últimos 3 meses a sua produtividade esteve reduzida a metade ou mais, devido às cefaleias? (sem contar com os dias que considerou na pergunta 1). |__|__|

3. Em quantos dias, nos últimos 3 meses, teve de deixar de fazer os seus trabalhos domésticos por causa das cefaleias? |__|__|

4. Quando ainda foi capaz de fazer os seus trabalhos domésticos, em quantos dias, nos últimos 3 meses, esteve a sua produtividade reduzida a metade ou mais devido à enxaqueca? (sem contar com os dias que considerou na pergunta 3). |__|__|

5. Em quantos dias, nos últimos 3 meses, teve de faltar a actividades com a sua família, actividades sociais ou de tempos livres por causa das cefaleias? |__|__|

Para ser preenchido pelo seu médico:
Por favor, some o total das pontuações das respostas às perguntas 1 a 5.

Pontuação MIDAS |__|__|

MIDAS_por-PT_nonMapi

© [illegible] 1997 (MIDAS VERSION [illegible])
